# Thrombopénie sévère chez un nouveau né de mère splénectomisé pour purpura thrombopénique idiopathique: Severe thrombopenia in an infant born to mother splenectomized for idiopathic thrombopenic purpura

**DOI:** 10.11604/pamj.2017.28.143.13880

**Published:** 2017-10-14

**Authors:** Sihame Lemouakni, Houria knouni, Amina Barakat

**Affiliations:** 1 Equipe de Recherche en Santé et Nutrition du Couple Mère-Enfant, Faculté de Médecine et de Pharmacie de Rabat, Université Mohamed V, Service de Médecine et Réanimation Néonatales, Centre Hospitalier Ibn Sina, Rabat, Maroc

**Keywords:** Thrombopénie, purpura thrombopénique auto-immune, grossesse, Thrombopenia, autoimmune thrombopenic purpura, pregnancy

## Abstract

La thrombopénie néonatale représente l’anomalie d’hémostase du nouveau-né la plus fréquente. Elle est définie par un chiffre de plaquettes inférieur à 150 000/mm^3^. Le risque de thrombopénie néonatale concerne 40% des nouveau-nés de mère ayant des antécédents de thrombopénie auto-immune et le risque de thrombopénie sévère est évalué à 10 à 15%. Nous rapportons à travers l’observation d’un nouveau né à J20 de vie de mère splénectomisé pour purpura thrombopénique idiopathique, la relation entre la gravité de la maladie maternelle et la gravité de la thrombopénie néonatale afin d’éviter le risque de survenue d’une hémorragie intracrânienne à l’origine de décès ou de séquelles neurologiques.

## Abstract (en)

Neonatal thrombopenia is the most common hemostatic abnormality in newborns. It is defined as a platelet count below 150.000/mm^3^. 40% of newborns to mothers with a history of autoimmune thrombopenia are at risk of developing neonatal thrombopenia while 10-15% of them are at risk of developing severe thrombopenia. We here report the case of a 20 days old newborn to mother splenectomized for idiopathic thrombopenic purpura in order to highlight the relationship between the severity of maternal disease and the severity of the neonatal thrombopenia and thereby to avoid the risk of intracranial hemorrhage resulting in death or neurological sequelae.

## Introduction

La thrombopénie néonatale représente l'anomalie d'hémostase du nouveau-né la plus fréquente. Elle est définie par un chiffre de plaquettes inférieur à 150 000/mm^3^. Le risque de thrombopénie néonatale concerne 40% des nouveau-nés de mère ayant des antécédents de thrombopénie auto-immune et le risque de thrombopénie sévère est évalué à 10 à 15% [1-3]. La conséquence principale de cette affection est la survenue d’hémorragie intracrânienne à l’origine de décès ou de séquelles neurologiques. L'objectif de notre travail était d'analyser la relation entre la gravité de la maladie maternelle et la gravité de la thrombopénie néonatale.

## Patient et observation

**Figure 1:**
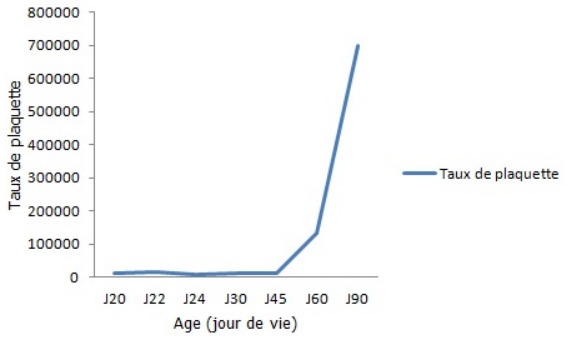
Evolution du taux de plaquette du malade

Nous rapportons l’observation d’un nouveau né de sexe masculin à J20 de vie, issue d’une grossesse menée à terme, accouchement par voie basse médicalisé, Apgar 10/10, poids de naissance 4kg. De mère âgée de 29 ans, suivie pour purpura thrombopénique idiopathique pour laquelle elle a été splénectomisée il y a 2 ans avec une thrombopénie à 66 000 /mm^3^ au cours de la grossesse. Le nouveau né a présenté à l’admission des ecchymoses cutanés avec une thrombopénie à 12000/mm^3^, pour laquelle il a bénéficié 48h d’immunoglobuline avec transfusion de plaquette et d’une ETF revenue normale. Evolution a été marquée par la disparition des ecchymoses avec un bilan de contrôle à 15000/mm^3^, le malade a reçus sa 2^ème^ dose d’immunoglobuline avec taux de plaquette à 8000/mm^3^. Devant la sévérité de la thrombopénie on a complété par un myélogramme et biopsie ostéomédullaire qui a éliminé une origine centrale puis il a été mis sous corticoïde .Le bilan réalisé 10 jours plus tard a montré la persistance de la thrombopénie à 10000/mm^3^ puis il a été revu à 2 mois de vie avec numération plaquettaire à 134000/mm^3^ et normalisation des plaquettes à 698000/mm^3^ après 3 mois (Figure 1).

## Discussion

Le purpura thrombopénique auto-immun est la cause la plus fréquente de thrombopénie néonatale. Environ 40% des nouveau-nés dont la mère a un purpura thrombopénique auto-immun sont thrombopéniques, mais seuls 10 à 15% d'entre eux ont une thrombopénie sévère avec un nombre de plaquettes inférieur à 50 x 109/L, à l'origine d'hémorragies intracrâniennes dans 1 à 3% des cas [1-4]. Cette anomalie résulte de la destruction des plaquettes par les anticorps maternels IgG qui traversent la barrière placentaire. Le nouveau-né peut présenter alors des signes cliniques hémorragiques plus ou moins graves en fonction de la sévérité de la thrombopénie. Certains auteurs suggèrent que la maladie auto-immune sévère est un facteur de risque de thrombopénie fœtale grave. Il a été rapporté aussi qu’un antécédent de thrombopénie ayant nécessité une splénectomie juste avant ou pendant la grossesse, expose le nouveau-né à un risque de thrombopénie sévère [5]. Une étude récente portant sur 64 cas de grossesses chez des femmes dont le purpura thrombopénique auto-immun était particulièrement sévère, 28 cas de splénectomies avant la grossesse et 17 cas de thrombopénie sévère pendant la grossesse trouve une thrombopénie néonatale sévère chez 57% des enfants [6]. Ceci est en accord avec les résultats d'une autre série de 55 femmes chez lesquelles les antécédents de splénectomie étaient corrélés avec une thrombopénie fœtale sévère [4]. Dans notre cas nous avons remarqué une corrélation entre la sévérité du purpura thrombopénique immunologique chez une mère splénectomisé et la survenue d’une thrombopénie sévère chez le nouveau-né. Dans la littérature, plusieurs équipes avec des approches différentes ont tenté d’évaluer quels enfants nés de mère avec un PTI sont susceptibles de présenter une thrombopénie sévère [7]. Certains auteurs ont évalué une attitude invasive en déterminant la mesure du chiffre plaquettaire au moment de l’accouchement par un prélèvement sanguin au scalp du fœtus [8, 9]. Mais cette approche donne souvent un chiffre faussement bas de plaquettes en raison d’une dilution par le liquide amniotique. Et la réalisation d’une ponction du sang du cordon ombilical in utéro s’associe à un taux de complications de l’ordre de 2-5% [4, 8, 10, 11]. Ainsi le risque d'atteinte fœtale est difficile à évaluer car il n'existe pas de corrélation entre le taux de plaquettes du nouveau-né et les différents paramètres maternels: taux de plaquettes maternel, taux d'IgG à la surface des plaquettes, taux d'anticorps anti-plaquettes sériques. Actuellement aucun facteur n’a été démontré prédictif pour le risque d’une thrombopénie néonatale sévère, le meilleur facteur prédictif semblant être la documentation d’une grossesse antérieure avec un enfant thrombopénique [7]. La sévérité de la thrombopénie associée ou non à des signes hémorragiques seront des éléments décisionnels pour la prise en charge thérapeutique. L’analyse de la littérature internationale montre que les seuils transfusionnels ne sont pas homogènes en fonction des pays et des pratiques. Ainsi, Gernsheimer et al. rapportent l’association transfusion plaquettaire et administration d’immunoglobulines intraveineuses (IgIV) à la dose d’1 g/kg par jour pendant 2 jours chez tous les nouveau-nés dont la numération plaquettaire à la naissance est inférieure à 30 G/L et chez ceux dont la numération plaquettaire est supérieure à 30 G/L mais présentant des saignements ou ayant des facteurs de risque de saignements [12]. Dans le cas où la numération plaquettaire se situe entre 30 et 50 G/L sans signe de saignements, seul un traitement par IgIV est alors mis en place. En France, les recommandations de bonne pratique place le seuil transfusionnel plaquettaire à 20 G/L.

## Conclusion

La thrombopénie néonatale d’origine immune n’est pas rare. Le problème principal dans la prise en charge de cette affection est celui de la prévention du risque hémorragique périnatal et donc de l'évaluation du risque de thrombopénie néonatale. Mais Aucun critère clinique ou biologique ne semble être prédictif de l'atteinte fœtale et de son intensité. Ainsi une collaboration et une prise en charge multidisciplinaire en cours de grossesse et à l'accouchement entre l'équipe obstétricale, le pédiatre et les hématologues sont indispensables et permettent une démarche parfois préventive et une prise en charge précoce pour éviter les séquelles neurologiques lourdes et la mortalité non négligeable.

## Conflits d’intérêts

Les auteurs ne déclarent aucun conflit d'intérêt.
